# Importance of Mediation against Addictive, Affective-Emotional, and Sexual Behavior in Adolescents. Educational System versus Associations

**DOI:** 10.3390/ijerph18031249

**Published:** 2021-01-30

**Authors:** Antonio Manuel Barbero-Radío, María Ángeles García-Carpintero Muñoz, José Rafael González-López

**Affiliations:** 1 Servicio de Salud del Ayuntamiento de Sevilla, Calle Fray Isidoro de Sevilla 1, 41009 Sevilla, Spain; abarbero@sevilla.org; 2 Department of Nursing, Faculty of Nursing, Physiotherapy and Podiatry, Universidad de Sevilla, C/Avenzoar nº6, 41009 Seville, Spain; joserafael@us.es

**Keywords:** adolescence, mediation, education and health promotion, socialization, qualitative research, educational system, associationism

## Abstract

This article analyzes health mediation among equals as an educational strategy against risk behaviors in young people from both the educational and associative systems in Seville (Spain), based on qualitative research, with the aim of assessing and comparing its impact in those areas. To this end, interviews with 49 professionals and 427 adolescents were conducted in discussion groups. Results acknowledge mediation as individual or group intervention accepted by young people as advice and information on health issues and conflict resolution, but also as a method for data collection in order to obtain a community health diagnosis. The educational system implements this strategy, but in associations it seems to work better, particularly in the psycho-emotional and sexual spheres. Unfortunately, intervention programs are usually discontinuous due to lack of resources and territorial variability. And this is why mediators’ support is highly valued by the target users, with preference for a male figure in the case of boys, and larger predisposition towards a female mediator in girls, except in cases where this agent has a special social relevance.

## 1. Introduction

The World Health Organization (WHO) defines adolescence—ages 10–19—as “the first stage of youth characterized by the maturation of human beings in their physical, sexual, psychological, and social dimensions” [1,2]. It is, primarily, a period of search for self-identity, and its end is marked by a psychological and social dimension regardless of the age of majority and cultural level achieved [3,4]. 

Health mediation (HM) is a health asset in “peer-education” strategies, optimized among equals when sharing any common characteristic: gender, age, or environment [5,6]. It is the training and support of some people by others of the same group or social rank, with special effectiveness with regard to the positive development model and the assets that promote it [7]. This strategy is carried out in the context of Health Promotion and Disease Prevention, in order to enhance the personal resources of the population [8]. It is the structured process of a group of agents that actively tries to inform, train, and support a similar population group on specific topics on which they have been previously trained [9,10,11].

Thus, in programs for the communication of health messages, peer agents become an important asset for effectiveness and efficiency, following the theory of socialization developed by J. Arnett [12,13] and of those that nourish the conceptual framework of mediation: Bandura’s Social Learning Theory [14], Rogers’ Diffusion of innovations, Tajfel and Turner’s Social identity theory, Fishbein and Ajzen’s Reasoned action [15], Freire’s Participatory education, and Hochbaum’s Health beliefs [16]. Thus, a group established as an educational resource leverages the implementation of mediation [17]. Consequently, there is a cascading effect of the message from its dissemination in the peer group [5,18]. In relation to the effectiveness of information and training actions, it depends on the mediators’ quality profile as well as on group counseling success, the messages coherence, the extent of community coverage, and the perseverance or periodicity of interventions [17,19].

The educational field at its different levels—school, secondary education, and university—represents the guarantee of strategy implementation. In fact, the infrastructures they possess and the value of education during the life process are crucial to train the population in the management and improvement of their health through the approach by communities [20]. This makes of schools and secondary education centers the ideal vehicle, as they provide the environment where children and adolescents spend a large part of their time [21], and during the evolutionary period of best knowledge, attitudes, and habits assimilation [22,23]. On the other hand, the university is considered an institution capable of reviewing its own internal systems, processes, and inherent culture, while fostering individual and organizational health and well-being [7,24]. In fact, there are networks of healthy educational centers in schools and universities both at national and international levels, such as the Schools for Health in Europe Network [25].

The intervention methodology within the school curriculum considers the need of implementing health-related issues [26,27], as well as providing teachers with a role of reference in health education with the help of external professional support, thus fostering the development of healthy skills and behaviors, facilitating information and participation channels within the educational center, posing stimulating challenges for all students, and focusing in active participation as a mediating agent, for example [18,28]. Moreover, it seeks to favor good relationships in school life, recognize and promote self-esteem and autonomy for a complete physical, mental and social wellbeing, promote coordination between the school and the family environment [29,30], and maximize community services as a tool for health promotion and education programs and activities, paying special attention to associations as educational resources [31,32].

Associationism is the active framework that serves as a theoretical support for group techniques based on its own definition: a mechanism that explains any human activity with the firm belief that the transformation of reality can be achieved through social engagement [33,34]. Thus, this resource is a good channel to organize and plan the actions previously considered by a group or entity, in order to improve the quality of life of the people in their neighborhood, city or town, from an inclusive health perspective [35]. The incidence of risk behaviors in the adolescent population could be improved through the associative movement [4], considering this as a space to be tutored by the Primary Health network [36]. Thus, any form of association can be part of the healthcare process in the context of healthy city, and, to this end, mutual aid groups are the best field of action [37].

Youth participation in associations pursues change and social transformation, though it faces some obstacles for its sustainability: financing, due to their dependence on the scarce public or private funds [38,39], and lack of physical spaces to meet [40]. Apart from these questions, there are some others that explain the shortage of youth participation, some of them related to changes in the traditional forms of socialization, where the media and social networks occupy most of the interest [34,41,42] And it is in the current cultural context where individualistic values emerge as time for oneself is highly valued, in contrast to the logic of solidarity and cooperation of associations [33,43]. Consequently, associationism becomes difficult in territories like suburbanized cities—even when they show a great amount of young population—increasing the risk of social exclusion, and without the civic commitment to avoid it [44,45].

Taking into account all of the above, the prevalence of youth risk behaviors [46], along with the difficulty which adolescents experience to internalize healthy messages based on the information and experience of professional teams and their own family, respectively [3], and the scarcity of research on the subject, the following study tries to shed some light on the impact of HM on young people, as well as its most effective field of action as an educational alternative trying to convey a health message. For example, through programs which converge in the Andalusian capital: “Young Form” (Andalusian Board) and “Education for Health in the regulated Educational Framework or outside of it” (Seville City Hall), both on voluntary demand.

## 2. Materials and Methods 

### 2.1. Design

The research questions guiding this study were “What do young people know about HM?” and “What knowledge do professionals have about it?” In order to answer them, a primary qualitative, descriptive cross-study [47] was carried out in two phases between 2012 and 2017, in the city of Seville, Spain.

The semi-structured interview as a tool for data collection allows the complete description of the experiences and thoughts of participants [48]. It started with five large open questions for all participants: ”What do you understand by HM?”, ”Which advantages and disadvantages can you identify with HM?”, ”What type of space do you consider is the most effective for HM to take place?“ ”Which topics are the most relevant for HM?”, and ”Who are the best recipients of HM?”. Sociodemographic data was collected taking into account the age and sex that the participants themselves indicated in the individual informed consents, together with the type of center: public or private, and system: educational or associative (signed by their tutor if they were not over the age of 18), such as the role and job position in the case of professionals. The research and its ethical considerations were explained in the consent itself, also in relation to the audio recording of the interviews.

### 2.2. Sample

The sample consisted of 476 participants, 45 semi-structured interviews, and 45 focus groups—with an average of 9 members—in which 427 young people between 14 and 24 years old participated. The study ages ranged from middle adolescence to the end of the second stage of youth (20–24 years), paying attention to maturation age as well as counting on the experience of those young people who were no longer adolescents—coming from secondary school centers and associations in Seville—joined 49 professionals from the health, social, and educational fields.

### 2.3. Sampling Strategy

The sample was intended to be as representative as possible regarding the city. In this sense, the willingness to participate without remuneration, favored its duration over time. Signed permissions were requested from educational centers and associations, as well as from young people through individual informed consent. This was accomplished under the same conditions of intervention, and in order to favor the response of young people it was agreed that it should be considered a curricular activity in health education. Thus, the group of young people was selected based on the fact that at the time of the research they were of legal age, they were in secondary education and/or actively participating in associations. Thus, for the study of HM in educational centers, there were 320 young people from 8 compulsory secondary education, Spanish Baccalaureate, and vocational training centers within the educational framework regulated in public, private or subsidized centers (privately managed educational centers subsidized by the public administration), with students living in the city. In addition, there were 107 young people outside the regulated educational framework members of 12 associations [49]. To form the discussion groups, the criterion followed was mixing groups of young people of the same age and/or environment (educational or associative mainly) for homogeneity [50]. As heterogeneity criteria, the area of the 11 municipal districts in which the city is segmented [51] and the socioeconomic level by synthetic index SI [46,52] were established, because the investigation was prolonged until having institutes and associations of all the districts and levels. These criteria addressed the level of studies, the level of qualification at work, the unemployment rate in the population aged 16 and over, and overcrowding factor: less than 16 m^2^ per person [53]. The synthetic index divided participants into 5 levels: SI 1: very high socioeconomic level, SI 2: high level, SI 3: average level, SI 4: low level, and SI 5: very low level [54,55]. Regarding the group of professionals who also signed the consent, it was made up of experts from the public and private sectors, with positions of responsibility in decision-making, and professionals considered top specialists in their field. For the rest, the investigation continued until the saturation of the speech, avoiding risk by avoiding theories, models and interpretations during data collection. In fact, the objective of phenomenology is to transform traditional ideas into perceived perceptions of what is investigated.

### 2.4. Measures Description

The total sample consists of qualitative characteristics or attributes: nominal dichotomous in the case of young people and polytomous variables in that of professionals, as it is shown in Table 1 and Table 2, respectively [56].

### 2.5. Data Compilation and Analysis 

The main researcher (author 1) collected the data. Both the interviews and the focus groups were recorded and transcribed verbatim; the average duration of the individual interviews was 30 min and that of the groups 60 min, approximately. And given that the intervention with young people was considered a curricular activity for health education, their response was higher than 98%. In this regard, those cases linked to both a group of the educational sphere and a group of the associative system, they could only choose one participant to intervene. For the rest, only a few could not participate due to a logistical issue, as they did not have the consent signed by their tutor, as well as due to non-integration with the group.

All researchers read all field notes and interview transcriptions several times, in order to gain an overall understanding of the content. The analysis of the data was based on the content of the texts resulting from the recordings, with the aim of developing concepts that contributed to explain, from a sustainable dimension, the exchange networks and the flows of meanings present in the educational and associative context [57]. This analysis continued by organizing descriptive labels, focusing on emerging or persistent concepts and similarities/differences in participants’ behaviors and statements. 

It is worth highlighting the contribution of the professionals who, although they did not participate in the analysis, were part of the triangulation process of various data sources in this case, honing the intrinsic deficiencies of the investigation to increase its validity. For this, several authors were contrasted, thus considering other points of view and optimizing conclusions [48]. The opinions incorporated were based on the same questions, paying special attention to key informants with expertise and influence, such as a Doctor in Evolutionary and Educational Psychology from the University of Seville, a Consultant and Researcher from the Area of Social Welfare, Citizenship and Communication of the Andalusian School of Public Health, and the President of the National and Ibero-American Association of Medicine and School and University Health.

The whole analytical process was carried out using the NVivo 11 software, after a first manual categorization according to the research objectives, for which the descriptive phenomenological method of Giorgi was applied [58,59]. This descriptive analysis entailed a reduction of data as part of a basic analysis to facilitate their management, so they were moved from the narrative to the descriptive field through segmentation into singular elements according to their relevance and significance for the research: textual units or verbatim (V) subjected to categorization and coding (see supplementary material Table S1), without any previous interpretations, and respecting the information obtained. Thus, the origin of each one was identified depending on whether the interview was individual “I”, or by group “G”, numbering each sector according to “n”, discriminating young people according to sex and age. With regards to professionals, key information—when applicable—as well their job profile were indicated. Subsequently, and in order to deconstruct the textual content, the syntactic, semantic, and pragmatic analysis of the verbatim was accomplished, providing an exact reproduction of the testimony. The form of the text, the type of language, the frequency, the categories order, and redundancy were observed through the syntactic analysis, and the different meanings given to the categories through a semantic analysis, besides whether or not there was a relationship between categories and sample profiles, characteristics of the subjects who spoke: a pragmatic analysis [57]. Finally, an analysis of the discourse in the whole text was carried out, trying to reproduce its meaning from the purely interpretive, hermeneutical perspective of the sample’s social discourse, with reference to its attributes regarding the HM in this case [56].

This research followed the criteria of The Consolidated Criteria for Reporting Qualitative Studies (COREQ). For the rest, the reliability audits on the part of the work supervisors and directives (there were three researchers and six reference key informants from among the 49 professionals interviewed) complete the techniques of transferability, credibility, dependency and confirmability as criteria of rigor and excellence of qualitative research. The transferability was examined through theoretical sampling as a strategy to maximize the amount of information collected in response to a non-probability sampling, in-depth description and extensive information collection since diversification allows easier comparison between different scenarios. Regarding the credibility of the study, it was examined through the triangulation of data as process confronting different data sources (visualizing reality from different perspectives of sex, age, types of centers and system -educational or associative- including a professional part, thus achieving a more complete result), extending the work looking for the isomorphism between the perceptions of the people investigated, checking it with the participants (systematic contrast of information with the agents, key informants and other professionals), persistent observation, peer comments (critical judgment of the interpretations made by the different researchers). With regards to dependability, it was examined by establishing review tracks, stating how the data were collected, how the informants were selected and how the data were analyzed. With respect to confirmability, it was examined using descriptors with a low level of inference, such as records faithful to reality from which the data were obtained (textual transcripts) and a reflection exercise with epistemological references that justify the interpretations.

### 2.6. Ethical Considerations 

The study was carried out with the authorization of the Biomedical Research Ethics Coordinating Committee (02/2016). For each of the cases, prior permission of the member entity was requested, as well as the written informed consent of each participant, confidential and subject to the Spanish Organic Law on Protection of Personal Data 15/99; current 3/18 on Personal Data Protection and guarantee of digital rights (State Agency Official State Gazette) [60,61].

## 3. Results

**Figure 1:**
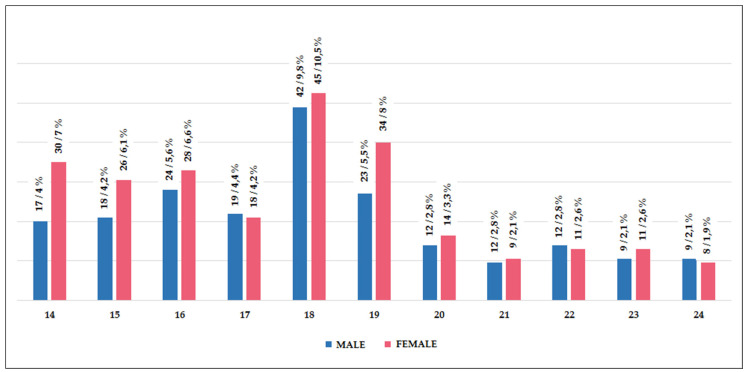
Frequency and percentage of young people according to their sex and age.

On the categorical or qualitative information included in the informed consents of the 427 youths among students and associates, sex, age, center and system were recorded with their corresponding features (including those arising during speech), and therefore it seemed logical to apply statistical descriptors only to age as the only quantitative variable in this case. Thus, the sociodemographic description of the sample of young people refers to 234 girls (54.8%) and 193 boys (45.2%), concentrating half of the total in those aged between 17 and 19. 287 of them belonged to public centers (67.21%) and 140 to private ones (32.79%); 320 to the educational system (74.94%), and 107 to associations (25.06%), without notable group differences with respect to the global analysis, as shown in the histogram—Figure 1, preferential analysis since all associates were in compulsory education. 

The central tendency statistics tool offers a report on the values of the sample of 427 young people with respect to the age variable in this case. Median: 18, Mean: 17.98, Range: 10, Variance: 7.481, Standard deviation: 2.735. The shape statistics check the drawing of the distribution of the data of the variable age in the graphic sample of young people. Asymmetry: 0.447, Kurtosis: −0.512.

With all these data compiled according to the age in the quoted sample, a slight positive asymmetry is interpreted in the sample, close to zero, compatible with a flared graphical distribution given the practical coincidence of the median, mean and mode. In addition, it is also platykurtic or negative pointing, because in the right and left tails of the figure there are more accumulated cases than in normal distributions. Finally, it is observed that 50% of the sample is concentrated in only three years; from 16 to 19, as well as that the ages between 25% and 50% of young people are more dispersed than between 50 and 75%.

With regards to sex and age, the major rate in almost all ages groups are girls; highlighting the difference between sexes at 14, 15 and 19 years. Otherwise, a slight majority of boys is observed at 17, 22 and 24 years only. 

Regarding the sample of professionals, it was aimed at top specialists in the field, regardless of their sex. In terms of age, most ranged between 45 and 55 years, which suggested experience without diminishing the experiences lived by the youth groups. The most frequent profile was the health professional, followed by the teacher: 19 (38.7%) and 17 (34.6%) respectively, while the main company was the regional administration: 29 (59.1%), and usual discourse from healthcare experience: 16 (32.6%), followed by the educational and associative spheres with the same number in each case: 14 (28.6%). 

In relation to inferences from the sample, the fact that women were more willing to do research was proportional to the number of young people willing to participate in the mediation strategy: 64 young people out of the total, 39 girls (60.9%), and 25 boys (39.1%). Otherwise, all educational centers in the sample were public or concerted, hegemonic characteristics of the majority of centers that offer secondary education in Seville: 111 (78.2%) of a total of 142, having participated in 8 (7.2%) of those that received public funds. Of these centers, 49 (34.5%) were publicly owned, having intervened in 6 (12.2%) of them. Nevertheless, the fact that there was a majority of teachers in the sample was due to the majority of secondary school centers included in it, followed in number by health professionals and health centers, respectively. On the basis of this, each professional focused on their expertise sphere. 

From the content analysis, a total of 33 categories were distinguished in three levels of segmentation, in relation to the ”concept”, ”benefit”, ”risk”, ”space”, ”theme”, and ”user” of HM. Of these 6. 27 emerging subcategories are derived: 21 + 3 + 3, as seen in Table 3. 

### 3.1. Are You Familiar with the “Concept” of HM?

HM is recognized as the educational method option for health improvement: “The voluntary action of approaching an agent who guides you through problem prevention, also in the health sphere.” (I 38 P IK, Psychopedagogue). Moreover, HM is recognized as the ideal alternative to the professional (V 1, V 2) or the family for young people (V 3, V 4), communicating a health message of a biological, psychological and/or social nature (V 5, V 6). In relation to the subcategory “Prevention of Health Problems” understood HM as strategy of education for health in adolescents, regardless of other acknowledgments of HM such as personal development or health diagnosis; out of a total of 120 interviews (75 focus groups, 44 individuals and 1 in couple), was part of the discourse in 108 including the 49 professionals and 415 young people out of a total of 427. Only 5 young people answered “not knowing” about mediation and by detachment from the system, while 7 declared that they acknowledge it but through other assets. 

HM ranges from global intervention through asset management policies (V 7, V 8) to group or individual intervention (V 9) through a peer mediator with attitude and aptitude for it, or health agents trained in an environment that can be either educational or social. This provides thematic information on the different levels of prevention (V 10), of mere advice or referral (V 11, V 12), and even social conflict resolution either in the field of human relations (V 13, V 14), or between the population and the health administration (V 15), taking into account cultural differences (V 16, V 17) “Health issues are dealt with in the social area and vice versa. They are not independent areas.” (I 29 P, Social Worker). This relationship lies in the deficit of emotional health due to collateral effects of population interaction (V 18). 

In fact, social mediation is considered part of HM (V 19, V 20), as it favors the psychological well-being of people (V 21), and this is acknowledged by older youth and professionals. In that sense it is bidirectional, as it also facilitates the collection of data for the detection of health needs or diagnosis in the community “Mediators know the real context and, therefore, they can design programs based on the history of the needs we have.” (G 3 P, Social Educator). In any case, it is always linked to any other individual or group mediation (V 22), as well as especially favored by the empathic quality of the mediator (V 23).

The reference to feedback stands out in professional discourses: “health system” and “associative” while, for the total sample, the best-known HM lies in the sender-receiver direction as thematic information and as advice or referral in portfolio of services, with special attention to the access protocol to existing resources. In the sample of the educational system, it is mostly used for conflict resolution in interpersonal relationships due to confrontation or isolation, and not so much as a result of problems with the administration (more common in the associative system and by specialized mediators). This is especially the case of private or subsidized centers, where social mediation is not related to HM. In this regard, it seems proved that people with less knowledge about the HM think that this is a strategy oriented to groups that exclude themselves from society (V 24), similarly to the conservative political sphere view, where there is great skepticism around its effectiveness, even identifying it. However, young people who say they “know nothing about it” tend to be from disadvantaged areas with low socioeconomic status and few educational resources. In spite of everything, the greatest knowledge and confidence about HM is observed in the cases of personal experience among those who had already received training and/or played the role of agent for mediation, as well as in those who have been receivers in the dynamics. 

Among young people, there seem to be no difference in the assessment of HM according to gender. While there are no differences among professionals regardless their profession and discourse profiles, but there are differences when it comes to the company, since the local administration, in collaboration with the university, is the most knowledgeable and major implementer of HM. This authority is followed by the Andalusian Youth Institute (IAJ), but its interventions are not so continuous and to a great extent depend on programs executed from the Public Health System. Despite this, the nurses in the health centers know the strategy, but do not have the means to carry it out fully, not even in coordination with other administrations. The lack of resources allocated to health mediation is a common standard.

### 3.2. Do You Consider HM a “Benefit”?

The strategy of HM itself seems key to a better access to the youth between this population and professionals (V 25, V 26). The informative group consultancies in the educational and associative frameworks serve as an exchange of experiences (V 27, V 28), but they improve with HM dynamics among peers: “The pedagogy already supports it and indicates that, with mediation processes, young people develop better.” (I 38 P IK, Psychopedagogue). This leads to a domino effect on the part of young people, even more so when an expert agent is involved, improving their knowledge on health issues: “It is crucial due to its amplification effect, starting with the young person who is a good mediator.” (I 23 P IK, Nurse). 

Sometimes young people do not dare to raise doubts to professionals out of shyness or ignorance, or simply because they do not even want to ask, and there HM becomes a support resource for the Health System: “Due to lack of knowledge, mediation is already interesting for them, and even more when helping with embarrassing topics.” (G 12 YW, 18 years old). In any case, no model based exclusively on information is effective or sufficient if it is not accompanied by participation and integration mechanisms (V 29) that transform the load of information related to messages from different sources (V 30, V 31) in knowledge and learning. In fact, it is of great value in young people excluded from the group (V 32) even empowering them (V 33), as well as in population at risk of social exclusion (V 34, V 35).

The group counseling model is stimulating for both the educational and the associative frameworks, as proved in reference to workshops and awareness days (V 36), and even more as an agent: “Young people who mediate discover new capacities. They are satisfied to help feeling like protagonists.” (I 23 P IK, Nurse). The group favors consultation with peers based on the trust that they share through the socializing process, which palliates the lack of communication with health professionals and families “Without the generation gap, the mediator is closer and therefore adolescents accept this role better. In a moment of full search for self-identity, they need alternative models to that of professionals and parents, to check them and compare them.” (I 20 P IK, Professor).

Each agent for mediation had the opportunity to meet many people in the environment and they usually receive more training than the rest of the group, specific practice that also contributes to their own background (V 37, V 38), which is recognized by peers: “We are lucky that they come to tell us about HM.”, “They care about our health.” (G 17 YW, 17 years old). The praising of mediation comes, particularly, from young people who were mediators, and from professionals (V 39). The latter also value the savings that the use of non-professional mediators represents for the administration of health assets “It is an asset at zero cost.” With the crisis, non-professionalization becomes a resource.” (I 20 P IK, Professor). 

### 3.3. Do You Consider HM a “Risk”?

Among the negative aspects of HM, the inaccurate messages due to a training deficit in mediator stand out: “Sometimes they are not very precise in what they communicate because maybe they are not well trained. However, we trust them and then it may cause us harm.” (G 34 YW, 17 years old). There is also the risk of possible misinterpretation due to misunderstanding by users (V 40). In relation to the latter, there are cases of poor active listening, which leads to a deficit in adherence to the strategy what they generally attribute to an incomplete profile of the mediator (V 41), regardless of the prejudices with more or less foundation related to the subject (V 42) or space of mediation; either in the regulated educational framework or in associationism (V 43), which may even lead to hostility towards agent “In case of conflict, proposals not favorable to one party may cause tensions with the mediator.” (G 36 YM, 16 years).

The lack of coordination may be also due to the gap between reality and the social health program that the mediator promulgates: “Sometimes what the mediator says does not coincide with the reality or group needs and the health programs.”, “The programs remain in the administrative field far from the knowledge of the technicians who work directly with the population.” (G 3 P, Social Educator). The latter is something common in areas of social transformation with low socioeconomic index, where they, and even the mediator, suffer from isolation from health services, which makes HM interventions difficult (V 44).

Finally, it should be noted that, despite promoting the participation of young people by means of mediation, there is a lack of interest in the health message since it is not among their priorities. Sometimes they do not get involved even if they verbally acknowledge the importance of the subject matter; hence the relevance of working on the level of self-esteem and assertiveness as the main protection factor: “Socio-emotional education favors health. With self-esteem, they will know how to say no to risky sexual behavior, drugs, etc.” (I 30 P, Psychologist). In fact, sometimes it is necessary to contain peer or partner pressure (V 45, V 46).

### 3.4. What Type of “Space” do You Think Is Ideal for HM to Take Place?

Although HM is considered a potential tool in any community space (V 47), participants state that it is not enough to address the target population, but that a minimum of comfort in conditions ranging from accessibility (V 48) to privacy (V 49) in order to facilitate communication (V 50) and becoming extremely important depending on the subject matter (V 51) and/or users’ profile (V 52). However, beyond the intimacy proportional to the effectiveness of the strategy, the educational framework is the most committed space to implement HM as a guarantor to reach all young people: “Intervening in Secondary Education schools, we guarantee that we reach all young people.” (G 3 P, Social Educator). The reason for this is the time that they necessarily spend in continuous interaction with the peer group: “They spend there many hours and they trust each other. In the street, maybe they get together or not.” (I 16 P, Sociocultural Animator).

The educational system is the natural context for learning (V 53, V 54), and highly favorable as it represents an update of constant knowledge in the face of diversity (V 55). However, mediation is inherent in the associative movement, and although of late onset and not fully encompassing, it presents higher levels of self-criticism and transformation possibilities: “With the capability of the educational system, a large number of young people is reached, while in associations the number is lower but with higher qualitative approach. There we can be more flexible and we adapt ourselves to the real needs of young people, so it works better.” (G 3 P, Social Educator).

However, the figure of the mediator does not usually change in the associative movement (V 56), which generates more confidence “Besides going to a placebo decide to attend, you assimilate everything better with the same mediator. This is not the same in high school.” (G 37 YM, 15 years old). In addition, mediation in associations is ideal for the promotion of values (V 57), with particular effectiveness in areas at risk of social exclusion (V 58). At the same time, it enjoys greater specificity (V 59, V 60), voluntariness, and better care in small follow-up groups (V 61, V 62), with interest proportional to the common goal that unites and encourages mutual aid “They have a common problem which makes them to empathize with one another, and even make them more united because they are few.” (G 59 YW, 16 years).

These results improve in health associationism as such (V 63). This advantage (V 64) prevails in the discourse of the associative system, but it is also recognized by the educational sample with proposals for collaboration between both systems: “If those from the associative network came to the high school center, everything would be better. I think they are more knowledgeable in many subjects.” (G 30 YM, 18 years old). All this occurs in the context of coordination with the administration through the enhancement of associationism in secondary school centers and universities, favoring their knowledge and the necessary subsequent increase of associations, and, at least, the number of volunteers or associates in the existing ones “This way, I think there would also be more volunteers in associations.” (G 36 YM, 16 years old). It would be something like a call for attention or recruitment from the Secondary School centers, to then launch mediation monitoring in the associative environment, with more effective minority groups which consolidates it as a health asset in the community: “I think that, after a presentation at an educational level, they get attention by making the association known.” (G 29 YW, 17 years old).

This collaboration and coordination is supported both by both young people (V 65) and the professional discourse of key informants in favor of the compatibility of both spaces “We do not have to choose, but rather put our energy in all areas where we can contact young people. Let us leverage the fact that everyone goes through a certain educational training, and let’s do it the same with the associative field; being more actively open makes it more effective for prevention. It is essential to add up.” (I 37 P IK, Professor).

Nevertheless, above the educational or associative systems, the success of the strategy is subordinated to the profile of the mediator (V 66), with the exception of the topic they deal with (V 67) beyond primary prevention (V 68). Otherwise, there is a preference for the associative movement in most of the sample, when ignoring the main difficulty of this system: the poor level of associationism among young people “There is little youth associationism, therefore, health must be taught in Secondary Education centers and in associations over the years.” (G 71 YW, 19 years). In fact, the decrease in the number of associations in general is given as a context. For this, leisure activities can be used as an engaging method: “For me it is like a hobby, and the safe environment makes it more effective. Everything is more efficient here.” (G 72 YM, 17 years old).

### 3.5. Which “Subjects” do You Consider Are More Likely Target of HM?

The most relevant topics for the sample are those related to affective and sex education (V 69), addictions prevention (V 70, V 71), prevention of traffic accidents (V 72), prevention of eating behavior disorders (V 73, V 74), and mental health. Risk behaviors in these topics are of special concern, and young people prefer to deal with them between equals, instead of turning to health professionals or family members “Some issues are more in demand than others. Perhaps in the sexual-affective context a peer advisor is more beneficial” (G 17 YW, 17 years old). “It is very important in emotional health and sexuality (inequalities included), but also in addictions. These are tricky issues that are best handled by mediators” (I 23 P IK, Nurse). However, any subject is susceptible to HM, regardless of the importance of the mediator’s profile (V 75).

### 3.6. Who Is the Ideal “User” for HM?

Interventions in Health Education are feasible at any age (V 76), although they assume their effectiveness to be carried out at an early age (V 77), showing more advantages and efficacy from puberty “It is especially effective in adolescents, although it can be done in any age group” (I 26 P, Counselor). “Young people adhere to the message because they trust the group, hence the importance of a mediating leader” (I 38 P IK, Psychopedagogue). They also demand the specificity of the agent in order to facilitate their empathy and identification as equals (V 78).

In relation to gender, they report a greater degree of involvement on the part of girls in general terms, with special attention to the message communication and dynamization (V 79). In this regard, adolescents prefer an agent of their same gender depending on the theme, especially boys “HM has an important gender factor. Some of them do not speak about the same topics with girls as with boys; even a female mediator does not seem to them the same as a male mediator (a biased vision in the process of identifying themselves with their gender and social identity” (I 5 P, Psychopedagogue).

## 4. Discussion

The highest participation of girls in the study as well as their testimonies in defense of mediation in the focus groups (potential users of the training strategy) and behavioral observations showed a greater predisposition to exercise mediation without compensation, at any age. This fact is mainly due to the differential cultural conditions for men and women, for which they still retain the traditional role of caregivers as a social construct on the basis of solidarity, cooperation, and mediation in society [62]. This sensitivity shared by many of the girls even suggests responsibility and a greater socio-educational and health commitment on their part. However, in associationism, male intervention was based on the involvement of entities in the public sphere; therefore, observing here a male majority only in political or sports organizations, which provide greater relevance or social prestige than the rest of the spheres researched in this sample, thus complying with Quiroga’s heterogeneity thesis in his research on sociability, associationism, and political practices [63].

The maximum concentration of young people occurred in the transition of middle and late adolescence, coinciding with adult thinking and with the consolidation of one’s identity and values. This favors any determination and positioning, as HM is an opportunity around the age of majority, when young people yearn to be visible according to the Selman Model about the development of social conscience, in order to join the community by achieving a greater role in it [5]. Regarding this attitude, a mere difference of 2 years can be a gulf in behavior and in the relationship with young people [46]; and the reason lies in the importance and number of physical, psychological, and social changes in such a short time; with special attention to brain development and self-esteem for positive adolescent development, as Oliva observed [7,24,64].

**Figure 2:**
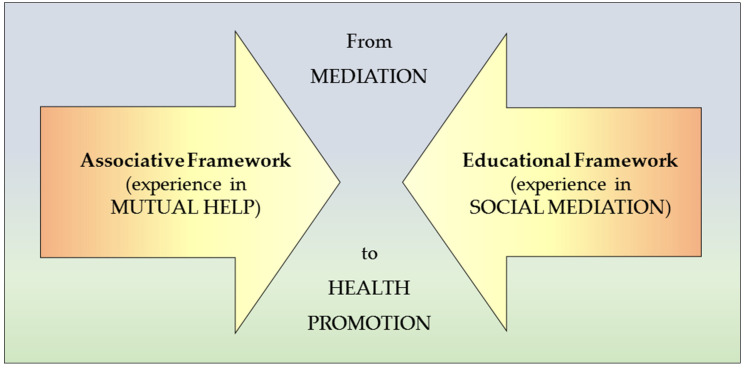
Confluence of systems for health promotion.

The entire sample showed their appreciation for the associative movement, detecting that most of the community entities that collaborated corresponded to the so-called third sector: private non-profit organizations that invest their profits into these entities to pursue their foundational objectives, taking control of their activities and with a marked degree of voluntary participation. All this seems to be the best complement to the educational system regarding HM, since it fulfills the associationism theory based on the experience developed by Hartley and Stuart from various philosophical contributions—Aristotelian to those by Locke and Hume—as well as attending to adjacency criteria due to proximity to the community, of similarity due to the common characteristics between cases, of contrast due to their own particularities, of frequency due to the possibilities of occurrence, and of recency effect due to the training chance provided by a mediator [65] between two systems that converge as we see in Figure 2, adding experience in favor of health promotion from the mediating strategy. 

The connection of the professional discourse with the company depended on the latter competences in the matter, so the discourse of the educational and health system rested in the autonomous administration that hires teachers and health workers, alluding to the competences and portfolio of services for this purpose. The latter were also dependent of the local administration, such as the professionals in the psychosocial field with respect to associations, since this profile and the local company are closer to the community, which is related to the greater mediating power of their agents.

Peer and/or group pressure is a facilitator of risky behaviors in the adolescent stage, particularly in relation to alcohol addiction—among other drugs—as the main predisposing factor for vehicle collisions, as there is also a lack of awareness in line with what they usually think: “Danger does not affect me”, “It is not likely to happen”, or “I can control it”. This is connected to the so-called fable of invincibility, in the egocentric context of the evolutionary psychology developed by Jean Piaget, as well as in the so-called personal fable by David Elkind [66] which involves the principle of autonomy of the adolescent and puts at risk the usual acceptance of the mediating agent.

Adolescents are the group with the greatest mediator for health potential regarding the prevention of bad habits due to the trust placed in their group. Hence the importance of training mediators among their own peers, in order to avoid situations of mistrust or embarrassment that may occur when dealing with their concerns with adults. In fact, peer mediators prove to be very efficient when dealing with risk behaviors in the sexual-affective sphere, including those of persistent male empowerment, and given that it is during adolescence that reasoning becomes more complex and attitude is most critical and reflective in a context of low self-esteem. Consequently, it is from this stage onwards that intervention is especially suitable, as it helps to assimilate the process of mediation together with the health message itself, in line with Bimbela’s theory of emotional leadership [67], which supports the capacities of the transmitting agent against the impulsiveness and thoughtlessness in younger ages, behavioral factors common to adolescents in full maturational process.

In any case, the educational system is the natural environment for the young, where they will also progress more and better while remaining healthy, as concluded by the 10th International Congress of School Medicine and Health, the 8th International Meeting of Experts in School and University Health, and the 29th edition of the Spanish Congress of Medicine and School and University Health, held in Cádiz in 2012, according to Sáez’s defense of school rights [68]. However, beyond this, potential health problems require an interdependent global society aware of the fact that the development of a response to the lack of interest in participation needs the promotion of a responsible global citizenship who believes that the development of some people is linked to development others, in line with Bandura’s vicarious learning theory [15]. In fact, it is important to claim a model of society where everyone has not only the right to participate, but also a solid structure of opportunities to accomplish so. This organizational model, as promulgated by Novel in favor of the HM [69], cannot be simply translated as such to world population, as it would need of an equitable redistribution of power, resources, and knowledge. Otherwise, a change in civic and participatory culture is necessary, as well as in the paradigms and structures of values and mechanisms for participation. From here on, any form of collective action that generates synergies and fosters processes of change and social development is accepted for its potential contribution to citizen awareness, to the emancipation of all people, to the fight for human dignity. It would be sufficient if citizens were more inclined to sacrifice part of their own interests for those of the community, once they become aware of what is happening to many others, everywhere. Then, institutions may or may not join this effort; in doing so, democracy will undoubtedly be strengthened.

As for the professionals, the testimonies of key informants stand out. However, all are experts and are explicit in the answers, while the young people often digress. In all, it is important to highlight the convenience of HM as an educational strategist with great potential for health protection, and as alternative to direct professional intervention through the prior training of mediators who can transmit the health message among equals; highly profitable among adolescents with attention to the fact that most recognize it although they hardly implement it in the absence of programs in this regard, since those in force from the administration, are lacking in resources and mainly deal with group counseling for the prevention of the disease. In order to achieve this, it would be interesting the collaboration of the centers and entities through the demand of the program in its entirety, as well as with the selection of leaders with a mediator profile to be trained, also in the associative and family sphere. In fact, what is the family role in this context? This ancient institution fulfills a crucial socializing function, not only transmitting current ideological values, but also interpreting cultural guidelines with particular adaptation to the children’s experience in the context of Y. Arnett’s theory of socialization [70], which includes peers and family as main sources of the process. In this sense, family is valued as a determining social agent in favor of a good emotional structure for the youth. However, far from underestimating it, research results place value on the educational framework -followed by the associative system, more effective- as the ideal context for risk behavior prevention and health promotion strategies together with the implementation of the mediating process, with the patronage of the so-called paternal law, present in the figure of the teacher and monitors in the associative environment, and regardless of their professional profile, degree, or specialization.

## 5. Limitations and Recommendations for Future Research

The main limitation of the study is the time elapsed between the data collection phases (until we met the authorization of the educational centers and associations with the intended location, we did not intervene) and time to reporting due to extensive analysis. For the rest, the sex of each participant was recorded, and the gender was observed, but no transgender analysis was considered. That said, when observing the slight sex polarization due to the greater number of women in the sample, the female discourse of the sample is highly valued, despite the homogeneous call to all young people. Otherwise, the results cannot be extrapolated, taking into account the particularity of the characteristics of this sample. However, outcomes can be taken as a reference despite their delimitation to the city of Seville, given the representativeness in the variety of social objects, centers, and entities. It would be a question of the transferability of results from a dense description of the intentional theoretical sampling. In any case, the prospective study relies on a higher profitability of Primary Care after reflection and enhancement of the HM, as well as from larger amounts to support its research by the administration. The extension of the study to other groups or areas, with special attention to stigmatized and/or socially excluded groups, as well as to a greater extent of the territory, will be highly valuable. 

## 6. Conclusions

Health mediation involves thematic information and/or health advice. It is bidirectional in that it facilitates data collection and health diagnosis in the community, being considered a health asset by professionals. Social mediation focuses on conflict resolution and therefore is part of health mediation because it contributes to the psychological well-being of people. Mediation is an accepted strategy for adolescents, more effective among peers. Their favorite contents are psycho-emotional and/or sexuality topics because they overcome the communication barriers they find with family and professionals, but also those related to the efficacy of addiction prevention. The educational space, as mandatory, offers a more relevant context to mediate with adolescents, but complementary to the associative one, that is much more effective because it is specific and voluntary nature, being more self-critical and working with smaller follow-up groups. There is a greater mediating sensitivity in girls, whereas in boys it only occurs in case of particular relevance of the agent, as well as same-gender mediators. Finally, associationism and mediation function as an extension of Primary Care, showing great advances in stigmatized groups and/or those in social exclusion. In fact, populations and areas with the lowest socioeconomic level are those in more need of mediation, even if they are the ones who know little or nothing about it. Programs discontinuity is due to both lack of resources and the great territorial variability.

## Figures and Tables

**Table 1:** Sample “Youth” (Y).

**Sex**	Woman (W)/Man (M)
**Age**	Adolescent/Youngster
**Center**	Private/Public
**System**	Educational/Associative
**Area**	Low synthetic index SI 1–2/High synthetic index SI 4–5
**Health agent**	Yes/No
**Avoidance or exclusion**	Group integration/No group integration
**Training**	Previous training in HM/No previous training in HM
**Experience**	Personal experience/No personal experience

**Table 2:** Sample “Professionals” (P).

**Professional profile**	Health, Social, Educational, or Socio-educational related
**Company**	Association, Secondary Education School, Health Center, City Council, Community Center, Health District, Delegation or Council, Permanent Education Center, and University
**Group/Discourse**	Associations, Educational System, Health System, and Key Informants (KI)

**Table 3:** Categories of analysis by segmentation.

1. Health Mediation
1.1. Concept
1.1.1. Not knowing 1.1.2. Prevention of health problems 1.1.3. Personal development 1.1.4. Health diagnosis
1.1.3.1. Advice
1.1.3.2. Conflict resolution
1.1.3.2.1. Human relations 1.1.3.2.2. Administration 1.1.3.2.3. Culture.
1.1.3.3. Link with social mediation
1.2. Benefit
1.3. Risk
1.4. Space
1.4.1. Special considerations 1.4.2. Associative and educational systems 1.4.3. Associative system 1.4.4. Education system 1.4.5. Health center 1.4.6. Public space 1.4.7. Any space
1.5. Thematic
1.5.1. Sexual-affective 1.5.2. Feeding 1.5.3. Traffic accidents 1.5.4. Addictions
1.5.5. Physical exercise 1.5.6. Medication 1.5.7. Mental health 1.5.8. Any subject
1.6. User
1.6.1. Youth 1.6.2. Any age

## Data Availability

Data is contained within the article or supplementary material.

## Supplementary Materials

The following are available online at https://www.mdpi.com/1660-4601/18/3/1249/s1, Table S1: Consolidated criteria for reporting qualitative studies (COREQ): 32-item checklist.

## Abbreviations

Transcribed verbatim of the interviews and the focus groups.

V 1	“Between us and the professional, someone should inform us... so we do not feel embarrassed when asking questions” (G 15 YM, 15 years).
V 2	“A mediator trained in health in contrast to other figures lacking that involvement“ (I 10 P, Head of Studies).
V 3	“Lean on other people than parents to find out about things that concern you” (G 6 YM, 17 years old).
V 4	“Facilitate students with guidelines which do not usually occur in formal education or in families” (G 2 P, Counselor).
V 5	“The process that is followed to achieve the best possible health; physical, biological, and social” (G 19 YW, 17 years).
V 6	“When we talk about health there are four major related areas: physical, emotional, social, and spiritual. The latter is crucial for the meaning of life, whether it has to do with religion or not. And the social area affects all the others, no doubt” (I 37 P IK, Professor).
V 7	“The processes set forward, by governments, for example, to ensure good health” (G 18 YW, 18 years).
V 8	1.—“Manage material resources” 2.—“Promote health, education in youth health, and others” (G 20 YW, 20 years).
V 9	“A health intermediary, apart from solving personal health doubts, must address the group” (G 21 YW, 18 years).
T 10	“When they have a health problem or may have it, people from the same environment guide them” (I 1 P, Social Worker).
V 11	1.—“Someone who helps you” 2.—“The process that allows you to receive help” (G 23 YM, 17 years).
T 12	“Helping a series of people, who are in a situation A, and move them to a more favorable B situation” (I 38 P IK, Psychopedagogue).
V 13	“Mediation between us. For example, if two are fighting someone intervenes to solve it” (G 17 YW, 17 years).
T 14	“It has to do with certain social agents” (I 20 P IK, Professor).
V 15	“The effective resolution or management of conflicts in health, either with the administration or the health staff” (I 34 P, Nurse).
V 16	“It is not only transmitting what health is to adolescents, but also, in hospitals, for example, there must always be a mediator to see if an intervention on someone can be carried out or not, according to their religion or culture” (G 45 YM, 21 years).
V 17	“For example, with immigrants, depending on the subject. Imagine dealing with sexuality in another language, and different culture” (I 30 P, Psychologist).
V 18	“A social mediator treats other needs different than health, but the pattern is the same, even with other objectives” (G 71 YW, 19 years).
V 19	“Social mediation is a part of health” (G 32 YW, 22 years).
V 20	“Health encompasses the social. The healthy person interacts with society, establishing social relationships” (I 31 P, Social Educator).
V 21	“The social refers to many problems within the field of health, but at a psychological level” (G 69 YW, 17 years).
V 22	“It is a communication channel between the population and the health workers, to detect and solve problems in real world” (I 6 P, Doctor).
V 23	“When a mediator is involved and close to the people, he/she will obtain information” (I 20 P IK, Professor).
V 24	“I have not heard about, but I know social mediation. The health issues must be addressed in the case of drug addicts or mentally ill people” (G 21 J F, 19 years).
V 25	“Mediation reaches where the professional does not; it detects and adapts a reliable message with an amplification effect” (G 7 YM, 18 years).
T 26	“The mediator is normalized; closer than the health professional and sees the problem with more objectivity” (I 4 P, Teacher).
V 27	“Also because of the workshops we do and other things that help too” (G 16 YW, 17 years).
V 28	“We have learned a lot being in class and in a small group. It went well, I think it has helped me” (G 17 YW, 17 years).
V 29	“Not with information sessions, but with more mediation dynamics between equals” (I 2 P, Teacher).
V 30	“For example, now with advertising, the food industry... the figure of the mediator is positive” (I 26 P, Counselor).
V 31	“In today’s society most of them are very lost and a guide helps them to commit to the healthiest scenario” (I 34 P, Nurse).
V 32	“Social health has to do with one’s health. It is good to interact, to engage with people” (G 41 YM, 21 years).
V 33	“In case of being a mediator, this is seen as important because what it does is useful; it has a positive effect” (I 22 P IK, Nurse).
V 34	“Mediation is important for social groups that are isolated, those which do not have or know any recourse” (I 5 YM, 18 years).
V 35	“I think there are other social groups at risk that do need a mediator to introduce health issues” (I 31 P, Social Educator).
V 36	1.—“They have helped us by clarifying doubts” 2.—“There were things I didn’t know and they informed me here first” (G 10 YF, 19 years).
V 37	“The mediator, apart from applying himself the information he learns, is also familiar with professional values” (G 5 YW, 17 years).
V38	“Learning with equals fosters communication skills, which improves with guidelines and experience” (I 3 P, Head of Studies).
V 39	1.—“A study indicated that mediators are highly recognized by their peers because they share problems with them and find help to solve them” 2.—“Both health and education professionals acknowledge the contribution of mediators” (I 23 P IK, Nurse).
V 40	“That the information is misunderstood” (G 47 YM, 15 years).
V 41	“Maybe the person who is providing mediation is not so good at it” (G 29 YW, 17 years).
V 42	“Depending on the health issue, like with sexuality-related ones, there will be those who out of shyness will remain in the dark before asking the mediator” (G 31 YF, 18 years).
V43	1.—“There is pressure in the current concept of school. It must provide comprehensive information with health promotion, but due to lack of time, they implement it in very physical terms, far from positive development” 2.—“Religious, voluntary activities, sports associations… allow transversal objectives of health promotion and development; but they are not known or used enough” (I 20 P IK, Professor).
V 44	“A possible risk lies in the time it takes for the mediator to inform the health services” (G 43 YW, 18 years).
V 45	“The environment will or will not influence your health habits. For example if you are in contact with alcoholics, you may end being one of them” (G 40 YW, 18 years).
V 46	1.—“The group thing can affect me psychologically: stress, anxiety due to pressure, etc.” 2.—“Psychosomatic” (I 32 P, Doctor).
V 47	“Any space is good adapting the resources. Even the street, as it is usual for kids” (I 27 P, Social Educator).
V 48	“A close place and confident approach, let’s say, instead of a remote or secluded place that does not make makes you feel comfortable” (G 39 YW, 17 years).
V 49	1.—“Let the mediator be alone with you without anyone else” 2.—“It doesn’t matter the place as long as it’s quiet” (G 33 YM, 18 years).
V 50	1.—“Where the young people speak at ease, freely” 2.—“In small groups, with more privacy and efficiency” (G 70 YW, 19 years).
V 51	“It depends. For example, sexuality in high school, but certain subjects are best avoided, even with the parents” (G 29 YW, 17 years).
V 52	“It depends on each young person. Perhaps, even though they may be interested, out of shyness they prefer to ask the mediator alone” (G 33 YM, 18 years).
V 53	“Right now they are learning, and they are going to remember more” (G 61 YM, 17 years).
V 54	“Education is crucial and high school centers must train in health; then, associations must specify” (I 27 P, Social Educator).
V 55	“The homogeneous sample of an association does not represent the total. At high school no one cares, and you see all kinds of stuff” (G 18 YW, 18 years).
V 56	“The mediator in the educational field stays for the educational stage; in associations this role can be extended” (I 33 P, Psychologist).
V 57	“The educational framework lacks experience. Association monitors mediate in health, incorporating values” (I 20 P IK, Professor).
V 58	“Where situation of social exclusion or the like makes the person feel a little out of place” (G 64 YW, 23 years).
V 59	“From the associations we can specify and help more. You work better” (G 3 P, Social Educator).
V 60	“Associations are more specialized and efficient, even though they are less in number. High School deals only with general topics” (G 55 YW, 18 years).
V 61	“You go because you want to. Also given its size, the same health issue is more effective in an association” (G 33 YM, 18 years).
V 62	1.—“A small group generates participation and interest” 2.—“A large group is more difficult to control” (G 70 YW, 19 years).
V 63	“For example, in an association of patients the health problem fosters unity even more, and they want to participate. It is not the same in Secondary Education centers” (G 65 YM, 17 years).
V64	“In associations, due to the voluntary nature, it is more effective. They want to listen; 100% of message and work reaches them” (G 30 YM, 18 years).
V 65	“Both: health education in schools and its subsequent development and focus on associations” (G 44 YW, 17 years).
V 66	“It has more to do with the mediator than with the place. The good one will reach out more people, but nevertheless something stays” (G 55 YW, 18 years).
V 67	“It depends on the subject. At secondary school centers, those topics that interest everyone, like sexuality” (G 70 YW, 19 years).
V 68	“Both are fine, but at Secondary School is needed to inform and prepare. The other is to solve what one already suffers” (I 32 P, Medical expert).
V 69	“Especially with affective and sex education” (I 22 P IK, Nurse).
V 70	“Not only in this case, also about drugs use” (G 17 J, 17 years).
V 71	1.—“On smoking”. 2.—“On alcoholism and drugs” (G 27 YW, 15 years).
V 72	“Also in the prevention of traffic accidents” (I 22 P IK, Nurse).
V 73	“On food (there are girls who throw their sandwiches at recess), among other issues that are not addressed” (I 2 P, Teacher).
V 74	“Anorexia” (G 39 YW, 17 years old).
V 75	“The mediation strategy can be used in almost all or all issues, I would say” (I 33 P, Psychologist).
V 76	“For people of any age because everyone’s health is important, anywhere” (G 40 YW, 19 years).
V 77	“It is best to raise awareness about health since childhood. Once you are a grown up with a set mind, it is more difficult” (G 49 YM, 17 years).
V 78	“You have to know how to adapt to the person who listens (including age), feel them to show closeness” (G 31 YW, 18 years).
V 79	“My needs as a younger person were not the same as when I became older; the mediator must take this into account” (G 71 UF, 19 years).
