# Supplementary material for: Importance of Mediation against Addictive, Affective-Emotional, and Sexual Behavior in Adolescents. Educational System versus Associations
Source: Int J Environ Res Public Health. 2021 Jan 30;18(3):1249. doi: 10.3390/ijerph18031249 (PMC7908402; doi:10.3390/ijerph18031249)
Supplement: Supplementary file 1 [file ijerph-18-01249-s001.pdf]

Table S1. Consolidated criteria for reporting qualitative studies (COREQ): 32-item checklist.

No	Item	Guide Questions/Description	Response
**Domain 1: Research team and reflexivity**			
Personal Characteristics			
1.	Interviewer/facilitator	Which author/s conducted the interview or focus group?	All the interviews were conducted by the author AMBR.
2.	Credentials	What were the researcher's credentials? E.g. PhD, MD	AMBR, MAGC and JRGL were PhD.
3.	Occupation	What was their occupation at the time of the study?	All authors were professor.
4.	Gender	Was the researcher male or female?	AMBR and JRGL are men, and MAGC is a woman.
5.	Experience and training	What experience or training did the researcher have?	All researchers had experience in carrying out qualitative research and the have been trained to conduct interviews.
Relationship with participants			
6.	Relationship established	Was a relationship established prior to study commencement?	No, there wasn't.
7.	Participant knowledge of the interviewer	What did the participants know about the researcher? e.g. personal goals, reasons for doing the research	Name, occupation, reasons for doing the research.
8.	Interviewer characteristics	What characteristics were reported about the interviewer/facilitator? e.g. Bias, assumptions, reasons and interests in the research topic	Name, occupation, contact method, reasons for doing the research.
**Domain 2: Study design**			
Theoretical framework			
9	Methodological orientation and Theory	What methodological orientation was stated to underpin the study? e.g. grounded theory, discourse analysis, ethnography, phenomenology, content analysis	Phenomenological and ethnographic approach with a discourse and content analysis.
Participant selection			
10.	Sampling	How were participants selected? e.g. purposive, convenience, consecutive, snowball	Convenience sampling.
11.	Method of approach	How were participants approached? e.g. face-to-face, telephone, mail, email	Face to face.
12.	Sample size	How many participants were in the study?	Total 427; 320 young high school students and 107 belonging to associations.
13.	Non-participation	How many people refused to participate or dropped out? Reasons?	11 for personal reasons.
Setting			
14.	Setting of data collection	Where was the data collected? e.g. home, clinic, workplace	The interviews were carried out in differents places.

15.	Presence of non-participants	Was anyone else present besides the participants and researchers?	No, it was not.
16.	Description of sample	What are the important characteristics of the sample? e.g. demographic data, date	Young high school students and / or, belonging to associations
Data collection			
17.	Interview guide	Were questions, prompts, guides provided by the authors? Was it pilot tested?	Yes, they were. / Yes, it was.
18.	Repeat interviews	Were repeat inter views carried out? If yes, how many?	No, they weren't.
19.	Audio/visual recording	Did the research use audio or visual recording to collect the data?	Audio recording.
20.	Field notes	Were field notes made during and/or after the interview or focus group?	Yes, they were (field notes).
21.	Duration	What was the duration of the inter views or focus group?	60 minutes.
22.	Data saturation	Was data saturation discussed?	Yes, it was.
23.	Transcripts returned	Were transcripts returned to participants for comment and/or correction?	Yes. Reviewed by 476 participants.
**Doman 3: Analysis and findings**			
Data analysis			
24.	Number of data coders	How many data coders coded the data?	Two (AMBR and MAGC).
25.	Description of the coding tree	Did authors provide a description of the coding tree?	Yes, we did.
26.	Derivation of themes	Were themes identified in advance or derived from the data?	Themes were derived using both methods.
27.	Software	What software, if applicable, was used to manage the data?	NUDIST Nvivo 11.
28.	Participant checking	Did participants provide feedback on the findings?	Yes. Reviewed by 49 informants.
Reporting			
29.	Quotations presented	Were participant quotations presented to illustrate the themes/findings? Was each quotation identified? e.g. participant number	Yes, there were. / Yes, there was.
30.	Data and findings consistent	Was there consistency between the data presented and the findings?	Yes, there was.
31.	Clarity of major themes	Were major themes clearly presented in the findings?	Yes, they were.
32.	Clarity of minor themes	Is there a description of diverse cases or discussion of minor themes?	Yes, there is.

Developed from: Tong, A. Sainsbury, P., and Craig, J. 2007. Consolidated criteria for reporting qualitative research (COREQ): A 32- ítem checklist for interviews and focus group. Int. J. Qual. Health Care 19: 349-357.
